# One- and Two-Particle Correlation Functions in the Cluster Perturbation Theory for Cuprates

**DOI:** 10.3390/ma16134640

**Published:** 2023-06-27

**Authors:** Valerii I. Kuz’min, Sergey V. Nikolaev, Maxim M. Korshunov, Sergey G. Ovchinnikov

**Affiliations:** 1 Kirensky Institute of Physics, Federal Research Center KSC SB RAS, Akademgorodok, 660036 Krasnoyarsk, Russia; kuz@iph.krasn.ru (V.I.K.); svinikolaev@sfu-kras.ru (S.V.N.); sgo@iph.krasn.ru (S.G.O.); 2 Siberian Federal University, Svobodny Prospect 79, 660041 Krasnoyarsk, Russia

**Keywords:** strong electronic correlations, cuprates, Hubbard model, cluster perturbation theory, 74.25.-q, 74.45.+c, 74.70.Xa, 74.20.Fg

## Abstract

The physics of high-$T_{c}$ superconducting cuprates is obscured by the effect of strong electronic correlations. One way to overcome this problem is to seek an exact solution at least within a small cluster and expand it to the whole crystal. Such an approach is at the heart of cluster perturbation theory (CPT). Here, we developed CPT for the dynamic spin and charge susceptibilities (spin-CPT and charge-CPT), with the correlation effects explicitly taken into account by the exact diagonalization. We applied spin-CPT and charge-CPT to the effective two-band Hubbard model for the cuprates obtained from the three-band Emery model and calculated one- and two-particle correlation functions, namely, a spectral function and spin and charge susceptibilities. The doping dependence of the spin susceptibility was studied within spin-CPT and CPT-RPA, that is, the CPT generalization of the random phase approximation (RPA). In the underdoped region, both our methods resulted in the signatures of the upper branch of the spin excitation dispersion with the lowest excitation energy at the (π,π) wave vector and no presence of low-energy incommensurate excitations. In the high doping region, both methods produced a low energy response at four incommensurate wave vectors in qualitative agreement with the results of the inelastic neutron scattering experiments on overdoped cuprates.

## 1. Introduction

The electronic and magnetic subsystems of a correlated material are strongly coupled. Classic examples are the cuprate high-$T_{c}$ superconductors, whose phase diagram contains antiferromagnetic (AFM), metallic, charge-density wave, and pseudogap states. Undoped cuprates corresponding to the half-filled system are AFM insulators. In this case, the spin dynamics correspond to the excitation of magnons and could be described by linear spin-wave theory. The physics, however, become more complicated once the holes are doped in the system. The holes are delocalized, and the AFM long-range order quickly disappears, allowing the new cooperative phases to arise. Recent resonant inelastic X-ray scattering (RIXS) studies have revealed magnon-like excitations in cuprates over a wide doping range from underdoped to heavily overdoped systems [1,2,3,4]. Moreover, momentum-dependent charge excitations have been observed in RIXS in the pseudogap phase of cuprates [5]. The spin and charge dynamics are coupled to the doping-dependent changes in the electronic structure observed in angle-resolved photoemission spectroscopy (ARPES) [6,7]. Furthermore, electronic excitations can be described by one-particle Green functions, and the calculation of the magnetic and charge excitations relies on two-particle response functions. Two-particle correlation functions provide important information about the ordered phases of a strongly correlated system. On the experimental side, inelastic neutron scattering allows one to directly observe the dynamic spin susceptibility, i.e., the two-particle spin–spin correlation function [8,9,10,11,12,13,14].

Cuprates have a quasi-two-dimensional structure, and the conductivity is provided by the electrons in the copper–oxygen plane. This was the reason for intensive studies involving two-dimensional models and, in particular, the effective low-energy Hubbard model, which provided a simple approach that retained the essential physics. However, a detailed study of the electronic and magnetic properties of cuprates requires a more realistic model, such as the three-band Emery model that includes both Cu-$d_{x^{2}-y^{2}}$ and O-$p_{x,y}$ orbitals [15].

There are a number of methods for studying the physics of strongly correlated systems and cuprates in particular. One subclass of these methods was developed to progress beyond a mean-field theory by relying on the numerical solution of a somehow simplified problem. These approaches include quantum cluster theories [16], extensions of the dynamic mean-field theories [17,18], quantum Monte Carlo approaches [19,20,21], and variational Gutzwiller-type methods [22]. Here, we focus on cluster perturbation theory (CPT). CPT is a straightforward way of calculating one-particle correlation functions, i.e., spectral functions [23,24]. The latter can be directly compared to ARPES data. CPT is one of a number of quantum cluster theories [16], and it is the most economical cluster method in terms of the necessary computation power. In CPT, the first step is the exact diagonalization (ED) of a small cluster. Therefore, the short-range correlations are treated precisely. In the second step, the intercluster interactions are included according to some kind of perturbation theory. There have been few attempts to expand CPT for the calculation of two-particle correlation functions. The authors of [25] used variational cluster approximation (CPT modified by a self-consistent procedure) to solve the Bethe–Salpeter equation for a two-dimensional Hubbard model. In [26], the spin susceptibility was calculated using the determinant quantum Monte Carlo method and CPT for the one-band Hubbard model. The authors of [27] extended CPT to compute two-particle correlation functions by approximately solving the Bethe–Salpeter equation for the one-band one-dimensional Hubbard model.

Here, we developed CPT for dynamical spin susceptibility, an approach we call *spin-CPT*. This was based on an explicit calculation of correlation functions via exact diagonalization with a subsequent extraction of the two-particle spectral lattice function in a CPT-like manner, similar to [26,28,29]. Then, we applied spin-CPT to a two-dimensional model of cuprates (the effective Hubbard model for the CuO plane based on the Emery model). We compared the results of the spin-CPT and CPT-RPA approaches [27,30]. The latter is a straightforward generalization of the random phase approximation (RPA), where the bare-electron Green functions forming the electron–hole bubble are replaced by those obtained by CPT. We showed that spin-CPT produced a low-energy response at four incommensurate wave vectors, which qualitatively agreed with the results of the inelastic neutron scattering on overdoped cuprates. Additionally, it allowed us to obtain a spectral intensity distribution resembling the upper branch of an experimentally observed hourglass dispersion in a wide doping range.

This paper is organized as follows: In Section 2, we discuss the model and approximations used for the study. In Section 3, the main results are presented. In Section 4, the results are discussed. In Section 5, the concluding remarks are provided.

## 2. Model and Methods

### 2.1. Model

The Hamiltonian of the Emery model [15] describes the copper $d_{x^{2}-y^{2}}$ and oxygen $p_{x,y}$ orbitals:

(1) $$\begin{array}{ccc} H_{pd} & = & \sum_{i,\sigma }\varepsilon_{d}n_{i\sigma }^{d}+\sum_{j,\sigma }\varepsilon_{p}n_{j\sigma }^{p}+\lambda U_{d}\sum_{i}n_{i↑}^{d}n_{i↓}^{d}+\lambda U_{p}\sum_{j}n_{j↑}^{p}n_{j↓}^{p}+\lambda V_{pd}\sum_{\langle i,j\rangle }n_{i}^{d}n_{j}^{p}\\ & + & t_{pd}\sum_{\langle i,j\rangle ,\sigma }{\left(-1\right)}^{P_{ij}}\left(d_{i\sigma }^{†}p_{j\sigma }^{}+H.c.\right)+t_{pp}\sum_{\langle \langle j,j^{\prime }\rangle \rangle ,\sigma }{\left(-1\right)}^{P_{jj^{\prime }}^{\prime }}\left(p_{j\sigma }^{†}p_{j^{\prime }\sigma }^{}+H.c.\right), \end{array}$$

where *i* denotes the copper sites, *j* denotes the oxygen sites, $n_{i(j)\sigma }$ is the number operator of holes on site i(j) with spin σ, $n_{i(j)}=n_{i(j)↑}+n_{i(j)↓}$, $P_{ij}$ and $P_{jj^{\prime }}^{\prime }$ are phase factors, and $d_{i\sigma }^{}$ ($p_{j\sigma }^{}$) destroys a hole with spin σ on a $d_{x^{2}-y^{2}}$ ($p_{x(y)}$) orbital and site *i* (*j*). The Coulomb interaction normalization constant λ is introduced for convenience to allow the variation of the interaction strength.

The following hopping parameters are used (here and below, all energy values are in eV): $t_{pd}=1.36$, $t_{pp}=0.86$. These are the hopping integrals of the multiband p−d model calculated for La${}_{2}$CuO${}_{4}$ [31] by projecting [32] the ab initio local density approximation electronic structure onto the Wannier function basis. The Coulomb parameters used here are $U_{d}=9$, $U_{p}=4$, and $V_{pd}=1.5$. The charge-transfer energy parameter is taken to have a typical value, $\varepsilon_{p}=3.6$ [33]. The one-electron energy of the copper *d*-orbital is set to zero, $\varepsilon_{d}=0$.

Since CPT has a cluster at its heart, the choice of this cluster is important. In a CuO plane, one can naturally distinguish a single element consisting of a copper ion surrounded by four oxygen atoms. The oxygen, however, belongs to the two neighboring cells. To overcome this difficulty, we used the canonical fermions of Shastry [34], following [35,36,37,38,39,40,41,42], thereby orthogonalizing the $p_{x,y}$ orbitals and ending up with a representation of a square lattice of orthogonal CuO${}_{4}$ cells:

(2) $$\begin{array}{ccc} H & = & \sum_{f,\sigma }\left[\sum_{\alpha }\varepsilon_{\alpha }n_{f\sigma }^{\alpha }-2t_{pd}\mu_{00}\left(d_{f\sigma }^{†}b_{f\sigma }^{}+b_{f\sigma }^{†}d_{f\sigma }^{}\right)\right]+\sum_{f,\alpha }\lambda U_{\alpha }n_{f↑}^{\alpha }n_{f↓}^{\alpha }+\sum_{f,\alpha <\beta }\lambda V_{\alpha \beta }n_{f}^{\alpha }n_{f}^{\beta }\\ & + & \sum_{f\neq g,\sigma }\left[-2t_{pd}\mu_{fg}\left(d_{f\sigma }^{†}b_{g\sigma }^{}+b_{f\sigma }^{†}d_{g\sigma }^{}\right)+2t_{pp}\nu_{fg}\left(a_{f\sigma }^{†}a_{g\sigma }^{}-b_{f\sigma }^{†}b_{g\sigma }^{}\right)\right.\\ & - & \left.2t_{pp}\chi_{fg}\left(a_{f\sigma }^{†}b_{g\sigma }^{}+b_{f\sigma }^{†}a_{g\sigma }^{}\right)\right]+H_{int}^{cc}, \end{array}$$

where *f* indexes a cell position (site); $\varepsilon_{\alpha }$ is the one-electron energy for an orbital index α, which takes values $\left\{a,b,d\right\}$, where *d* is the copper orbital and *a* and *b* are the cell oxygen orbitals with the energies $\varepsilon_{a}=\varepsilon_{p}+2t_{pp}\nu_{00}$ and $\varepsilon_{b}=\varepsilon_{p}-2t_{pp}\nu_{00}$, respectively; the operator $d_{f\sigma }^{}$ annihilates a hole with spin σ on a copper orbital at site *f*; operators $a_{f\sigma }^{}$ ($b_{f\sigma }^{}$) annihilate a hole with spin σ on an oxygen orbital *a* (*b*) at site *f*; $n_{f\sigma }^{\alpha }$ are the number operators; and $n_{f}^{\alpha }=n_{f↑}^{\alpha }+n_{f↓}^{\alpha }$. The Wannier coefficients μ, ν, and χ and other constants related to the parameters of Equations (1) and (2) are the same as in [38,40,41,42]. In particular, the effective Coulomb interactions and the original interactions are related as follows: $U_{a}=U_{b}\approx 0.21U_{p}$, $V_{ad}=V_{bd}\approx 0.91V_{pd},U_{ab}\approx 0.17U_{p}$. Finally, $H_{int}^{cc}$ refers to all non-local interaction terms, including three-site and four-site ones, which did not have a significant influence on the results obtained using Equation (2) and are omitted hereafter.

**Figure 1:**
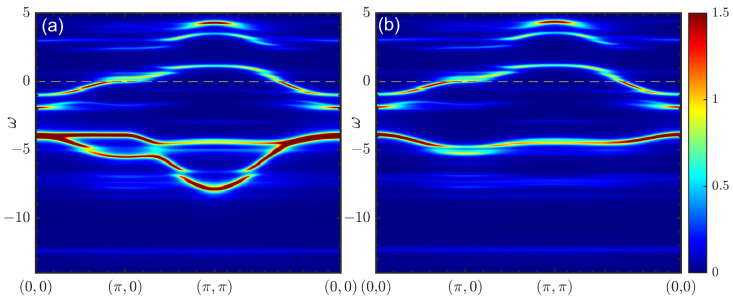
The electronic spectral function calculated via CPT using a 2×2 cluster for the Hamiltonian (2) including (**a**) and excluding (**b**) the *a* orbital. Lorentzian broadening at η=0.1 was used here and below.

Such a cell representation is especially useful when one is interested in the low-energy properties. Since only *b* orbitals are directly connected by the hopping processes with *d* orbitals, they should have a negligible effect on the low-energy part of the electronic structure. On the other hand, at a high doping level, *a* orbitals might become important [36]. In Figure 1, a comparison of the spectral functions obtained with and without the *a* orbital is shown for the overdoped case with a doping level of p=0.25 using a 2×2 cluster consisting of four orthogonal cells. A distinguishable difference was observed only 2 eV below the Fermi level within the bands dominated by the oxygen spectral weight [43]. Since the needed computational power rose with the number of possible states, which increased with the number of orbitals considered, we could increase the cluster size once we removed one of the orbitals. Thus, in the main part of this paper, we omit the *a* orbitals. This allowed us to extend the cluster size within CPT to 3×3, comprising nine orthogonal cells, with moderate computational effort.

### 2.2. Methods

The central object of our calculations is the transverse part of the dynamic spin susceptibility $\chi_{\alpha \beta ,\gamma \delta }$(q, Ω). It is defined as an analytical continuation of the Matsubara two-particle spin–spin correlation function

(3) $$\chi_{\alpha \beta ,\alpha^{\prime }\beta^{\prime }}\left(q,i\omega_{n}\right)=\int_{0}^{1/T}d\tau e^{i\omega_{n}\tau }\langle T_{\tau }S_{\alpha \alpha^{\prime }}^{+}\left(q,\tau \right)S_{\beta^{\prime }\beta }^{-}\left(-q,0\right)\rangle .$$

where $S_{\alpha \alpha^{\prime }}^{+}$ and $S_{\beta^{\prime }\beta }^{-}$ are the spin raising and lowering operators, respectively; q is the wave vector; $\omega_{n}$ is the Matsubara frequency; α, β, $\alpha^{\prime }$, and $\beta^{\prime }$ are the orbital indices; $T_{\tau }$ is the ordering operator over Matsubara (imaginary) time τ; and *T* is the temperature in the energy units. Averaging is carried out with the fully interacting Hamiltonian. By using the Wick’s theorem, we obtain the zeroth order approximation for the spin susceptibility in terms of the one-particle Green function $G_{\alpha \beta \sigma }\left(p,i\omega_{m}\right)$ for an electron with spin σ:

(4) $$\chi_{\alpha \beta ,\alpha^{\prime }\beta^{\prime }}^{\left(0\right)}\left(q,i\omega_{n}\right)=-T\sum_{\omega_{m},p}G_{\alpha \beta ↑}\left(p,i\omega_{m}\right)G_{\alpha^{\prime }\beta^{\prime }↓}\left(p+q,i\omega_{n}+i\omega_{m}\right).$$

The analytical continuation $i\omega_{n}\to \Omega +i\eta$ includes the positive infinitesimal parameter η that we use to introduce Lorentzian broadening in the course of the numerical calculations. Since we considered the paramagnetic phase, the spin index was omitted.

The physical spin susceptibility is calculated on real frequencies Ω as the sum over all orbital indices coinciding at vertices:

(5) $$\chi \left(q,\Omega \right)=\frac{1}{2}\sum_{\alpha ,\beta }\chi_{\alpha \alpha ,\beta \beta }\left(q,\Omega \right).$$

Analogously to the spin correlation function (3), the charge correlation function is defined as

(6) $$\Pi_{\alpha \beta ,\alpha^{\prime }\beta^{\prime }}\left(q,i\omega_{n}\right)=\int_{0}^{1/T}d\tau e^{i\omega_{n}\tau }\langle T_{\tau }\rho_{\alpha \alpha^{\prime }}\left(q,\tau \right)\rho_{\beta^{\prime }\beta }\left(-q,0\right)\rangle .$$

where $\rho_{\alpha \alpha^{\prime }}$ is the particle density operator. The physical charge susceptibility on real frequencies Ω is calculated as

(7) $$\Pi \left(q,\Omega \right)=\frac{1}{2}\sum_{\alpha ,\beta }\Pi_{\alpha \alpha ,\beta \beta }\left(q,\Omega \right).$$

#### 2.2.1. CPT

The electronic structure of a two-band model is studied using CPT, as discussed in detail in [23,24]. There are two steps in this approach. The first is the exact solution for the small cluster usually achieved by the exact diagonalization of the model Hamiltonian. Thus, the lattice is partitioned into a superlattice of clusters with a new translational order of an artificial origin. The second step is the reconstruction of the whole lattice by adding intercluster interactions, which is carried out in a perturbative manner.

For the Hamiltonian (2), we calculate the electronic spectral function

(8) Aαβ(k,ω)=−1πImGαβ(k,ω+iη),

where $G_{\alpha \beta }\left(k,\omega \right)$ is the electronic Green function on the real frequencies ω, k is a wave vector, α and β are the orbital indices, and $\eta \to 0^{+}$. The trace of (8) provides the total (physical) spectral function $A\left(k,\omega \right)=\sum_{\alpha }A_{\alpha \alpha }\left(k,\omega \right)$. CPT allows one to calculate A(k,ω) within a small cluster and an arbitrary momentum resolution; it is also characterized by a fast convergence of results with an increasing cluster size [44]. Here, the intracluster interactions are taken into account by means of the ED of a cluster with open boundary conditions using the Lanczos method, while the intercluster hoppings are treated perturbatively. This approach allowed us to include the short-range (intracluster) correlations explicitly. The calculations are carried out at a zero temperature using a square cluster consisting of nine cells (sites).

The main CPT equation is

(9) $$\hat{G}{\left(\tilde{k},\omega \right)}^{-1}={\hat{G}}^{c}{\left(\omega \right)}^{-1}-\hat{T}\left(\tilde{k}\right),$$

where all square matrices have dimensions $L\times N_{c}$; *L* is the number of orbitals, which in our case was equal to two; $N_{c}$ is the number of sites within the cluster; $\tilde{k}$ is a wave vector defined within the cluster Brillouin zone; ω is a frequency; ${\hat{G}}^{c}\left(\omega \right)$ is the exact local (intracluster) propagator; and $\hat{T}\left(\tilde{k}\right)$ is a Fourier transform of the hopping matrix. The translational invariance is restored in CPT as

(10) $$G_{\alpha \beta }\left(k,\omega \right)=\frac{1}{N_{c}}\sum_{i,j}e^{-i\left(r_{i}-r_{j}\right)k}G_{\alpha \beta ,ij}\left(k,\omega \right),$$

where i(j) is an intracluster site index, and $r_{i(j)}$ is a corresponding radius vector.

#### 2.2.2. CPT-RPA

CPT can be used to improve the calculation of the dynamic spin susceptibility χ(k,Ω) within RPA by replacing the bare electron–hole bubble, $\chi^{\left(0\right)}\left(k,\Omega \right)$, with that calculated using CPT Green functions. This allows one to include self-energy corrections over the vertex renormalization of RPA. This methodology is discussed in detail in [27,30]. Thus, given an RPA vertex, the obtained magnetic structure is determined by the electronic spectrum. Instead of the “bare” susceptibility, the electron–hole bubble is calculated via the cluster electronic spectral functions [30]:

(11) $$\chi_{\alpha \beta ,\gamma \delta }^{c(0)}\left(k,\Omega \right)=-\sum_{q}\int \int d\omega^{\prime }d\omega^{\prime \prime }A_{\alpha \beta }\left(q,\omega^{\prime }\right)A_{\gamma \delta }\left(k+q,\omega^{\prime \prime }\right)\frac{f\left(\omega^{\prime }\right)-f\left(\omega^{\prime \prime }\right)}{\omega^{\prime }-\omega^{\prime \prime }+\Omega +i\eta },$$

where f(ω) is the Fermi function, and η is the positive infinitesimal.

The transverse dynamic susceptibility within CPT-RPA is calculated as [45,46]

(12) $$\chi_{\alpha \beta ,\gamma \delta }\left(k,\Omega \right)=\chi_{\alpha \beta ,\gamma \delta }^{c(0)}\left(k,\Omega \right)+\sum_{\alpha^{\prime },\beta^{\prime },\gamma^{\prime },\delta^{\prime }}\chi_{\alpha \beta ,\alpha^{\prime }\beta^{\prime }}^{c(0)}\left(k,\Omega \right){\bar{U}}_{\gamma^{\prime }\delta^{\prime }}^{\alpha^{\prime }\beta^{\prime }}\chi_{\gamma^{\prime }\delta^{\prime },\gamma \delta }\left(k,\Omega \right).$$

A vertex $\bar{U}$ including another renormalization constant $\lambda^{\prime }$ that will be discussed below is defined as $\bar{U}=\lambda^{\prime }U$, where the nonzero components of the RPA vertex U are $U_{\alpha \alpha }^{\alpha \alpha }=U_{\alpha }$ and $U_{\alpha \beta }^{\alpha \beta }=V_{\alpha \beta }$, with α≠β [45,46]. The particle–hole bubble $\chi^{c(0)}\left(k,\Omega \right)$ was calculated in this study using the CPT spectral function as in [30].

Dynamic charge susceptibility Π(q,Ω) is obtained through CPT-RPA by replacing the vertex U in the RPA Equation (12) with the vertex −V, where $V_{\alpha \alpha }^{\alpha \alpha }=U_{\alpha }$, $V_{\beta \beta }^{\alpha \alpha }=2V_{\alpha \beta }$, $V_{\alpha \beta }^{\alpha \beta }=-V_{\alpha \beta }$ [45].

#### 2.2.3. Explicit Calculation of the Two-Particle Correlation Function (Spin-CPT and Charge-CPT)

An alternative to the RPA-like approach is to calculate the two-particle correlation function directly without the intermediate steps of finding the one-particle Green function and building a spin or charge correlation function out of it. Here, we calculated the right side of Equation (3) in a CPT-like manner and refer to this approach as *spin-CPT*. One-particle CPT takes the exact intracluster correlations and treats the intercluster ones approximately, for example, within the Hubbard-I approximation for the Green functions built on the Fermi-type Hubbard operators [47,48,49,50]. A straightforward application of this ideology with respect to Bose-type Hubbard quasiparticles forming the spin excitations leads to an approximation in which intercluster interaction does not enter the Green function calculation procedure, since no spin–spin interaction terms to be taken into account by the generalized Hubbard-I approximation are present in the Hamiltonian (2). Moreover, the intercluster vertex corrections have been shown to have no crucial effect on such cluster calculations [26,28,29]. Thus, the whole procedure is completed in two steps and is identical to the approach implemented in [26,29]. At first, the transverse spin susceptibility is calculated by the ED within the cluster via the following equation:

(13) $$\chi_{\alpha \beta ,ij}^{c}\left(\Omega \right)=\sum_{\mu }\left[\frac{\langle 0|S_{\alpha ,i}^{+}|\mu \rangle \langle \mu |S_{\beta ,j}^{-}|0\rangle }{\Omega -\left(E_{\mu }-E_{0}\right)+i\eta }-\frac{\langle 0|S_{\beta ,j}^{-}|\mu \rangle \langle \mu |S_{\alpha ,i}^{+}|0\rangle }{\Omega +\left(E_{\mu }-E_{0}\right)+i\eta }\right],$$

where $S^{-}$ and $S^{+}$ are the spin ladder operators, and $|0\rangle$ and $|\mu \rangle$ are the ground and excited states, which are obtained in the Lanczos approximations. Then, the translational invariance for the whole lattice is restored analogously to Equation (10):

(14) $$\chi_{\alpha \beta }\left(k,\Omega \right)=\frac{1}{N_{c}}\sum_{i,j}e^{-i\left(r_{i}-r_{j}\right)k}\chi_{\alpha \beta ,ij}^{c}\left(\Omega \right).$$

The dynamic charge susceptibility Π(q,Ω) can also be calculated in a similar manner on a cluster as follows:

(15) $$\Pi_{\alpha ,\beta ,i,j}^{c}\left(\Omega \right)=\sum_{\mu }\left[\frac{\langle 0|n_{\alpha ,i}|\mu \rangle \langle \mu |n_{\beta ,j}|0\rangle }{\Omega -\left(E_{\mu }-E_{0}\right)+i\eta }-\frac{\langle 0|n_{\beta ,j}|\mu \rangle \langle \mu |n_{\alpha ,i}|0\rangle }{\Omega +\left(E_{\mu }-E_{0}\right)+i\eta }\right],$$

where $n_{\alpha ,i}$ is the number of the particle operator. Since such an approach allows one to calculate the charge correlation function directly, we refer to it as *charge-CPT*.

## 3. Results

**Figure 2:**
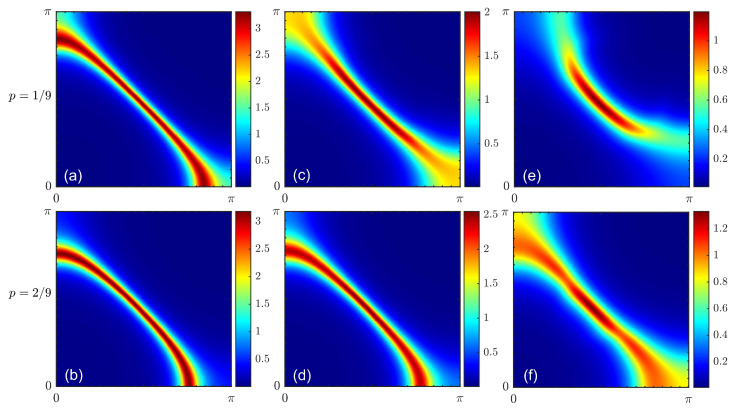
The electronic spectral function at the Fermi level for doping with p=1/9 (**a**,**c**,**e**) and p=2/9 (**b**,**d**,**f**) obtained using the Coulomb interaction normalization constants λ=0 (**a**,**b**), λ=0.3 (**c**,**d**), and λ=1 (**e**,**f**).

**Figure 3:**
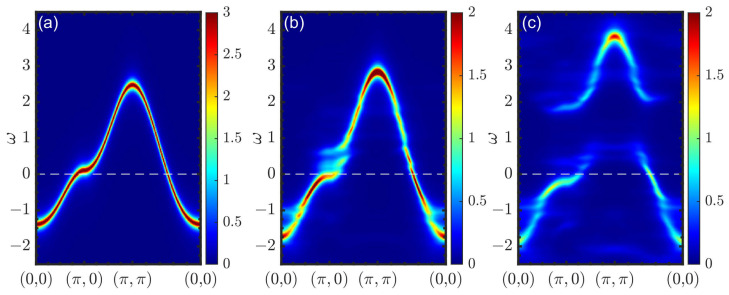
Energy–momentum distribution of the electronic spectral function calculated for doping at p=1/9 using the Coulomb interaction normalization constants λ=0 (**a**), λ=0.3 (**b**), and λ=1 (**c**).

To determine the changes in the low-energy electronic structure with doping and interactions, we started with the calculation of the Fermi surface for the CuO model (2). Two doping values corresponding to an integer number of electrons per cluster are shown in Figure 2, where we present the spectral function at the Fermi level. We later refer to such plots as the ‘Fermi surface plots’, although they shown neither the Fermi surface nor the Fermi contour itself. However, they are useful, since one can straightforwardly compare them to ARPES plots, which commonly show the distributions of the spectral weight in the narrow energy window near the Fermi level. The momentum–energy distribution of the spectral weight is shown in Figure 3. This distribution represents the band structure corresponding to the Fermi surface plots of Figure 2 for a doping level of p=1/9. Note the formation of the Hubbard subbands and the pseudogap at the Fermi level with the increasing interaction. Without Coulomb interactions (λ=0), the Fermi surface was electron-like. This was a direct consequence of the choice of band structure parameters. This shape did not agree with the ARPES results that showed a hole-like Fermi surface and even Fermi arcs at a low doping level *p* [6,7]. However, once we included the electronic correlations induced by the Coulomb interaction by increasing the parameter λ, the Fermi surface changed dramatically at a small *p*. For λ=0.3, it was already hole-like. The spectral weight decreased from the nodal to the antinodal direction, which was a signature of the pseudogap. Qualitatively, the obtained Fermi arc distribution of the spectral wight agreed with the results of the dynamic variational Monte Carlo approach [51]. On the contrary, the correlation effects were small at a high doping level. The Lifshitz transition occurred in between the presented values of doping at p≈0.13. The overall evolution according to the doping amount qualitatively agreed with the ARPES data [6,7] and the results of the mean-field strong coupling approach [52,53], where the appearance of two quantum phase transitions associated with the Fermi surface topology change from small hole pockets to a large hole-like and then electron-like Fermi surface was demonstrated. For a full-strength interaction, λ=1, the pseudogap was clearly visible at a low doping amount, while the electron-like Fermi surface at p=2/9 was affected only slightly. This time, the change in topology appeared at p≈0.17.

**Figure 4:**
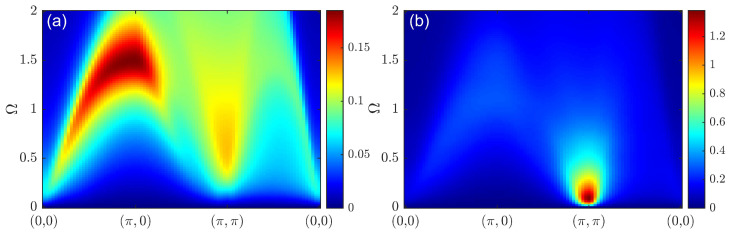
Dynamic spin susceptibility χ(q,Ω) calculated via CPT-RPA with a 3×3 cluster for doping at p=1/9 and Coulomb interaction renormalization constants $\lambda =\lambda^{\prime }=0$ (**a**) and $\lambda =\lambda^{\prime }=0.3$ (**b**).

**Figure 5:**
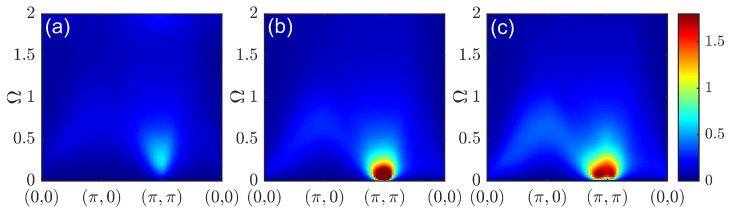
Dynamic spin susceptibility χ(q,Ω) calculated via CPT-RPA with λ=1 and $\lambda^{\prime }=0.75$ for doping at p=1/9 (**a**), p=1/6 (**b**), and p=2/9 (**c**).

Now, we turn to the CPT-RPA results, which demonstrate how the spin spectrum was affected by the one-particle processes entering the particle–hole bubble $\chi^{c(0)}\left(k,\Omega \right)$ through the spectral functions and enhanced by the RPA vertex. The dynamic spin susceptibility under CPT-RPA with a Lorentzian broadening of η=0.1 is presented in Figure 4 and Figure 5. At a low doping level and relatively weak interaction strength (Figure 4), changes in the electronic structure such as the emergence of the pseudogap resulted in the formation of a response resembling a spin wave spectrum. The lowest energy region was dominated by a contribution of the spectral weight at the antiferromagnetic wave vector due to the presence of the short-range antiferromagnetic correlations dominating the electronic spectrum.

**Figure 6:**
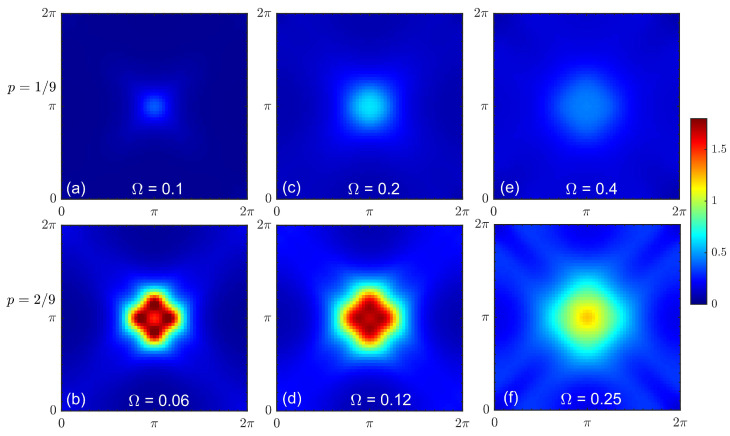
Constant energy cuts of the dynamical spin susceptibility calculated under CPT-RPA with λ=1 and $\lambda^{\prime }=0.75$ for doping at p=1/9 (**a**,**c**,**e**) and p=2/9 (**b**,**d**,**f**) and an energy of Ω (corresponding value is shown in each panel).

Figure 5 shows the doping dependence of the magnetic spectrum under CPT-RPA for a strong interaction. Here, the Coulomb interaction included in CPT and $\chi^{c(0)}\left(k,\Omega \right)$ was at full strength with λ=1. Parameter $\lambda^{\prime }$, which controlled the strength of the interactions entering the RPA vertex, however, was taken to be less than unity to avoid magnetic instability at finite doping, i.e., the divergence of the real part of the zero-frequency RPA spin susceptibility under CPT-RPA. We adjusted $\lambda^{\prime }$ so that the system was in the vicinity of instability, which appeared first at the Lifshitz transition, where the particle density at the Fermi level was maximal (p≈0.17 in the present case). In the pseudogap regime, the response was again somehow similar to the spin wave spectrum; however, it was highly damped. The energy of the (π,π) spectral weight maximum tended to zero at the Lifshitz transition point; see Figure 5b. A qualitative change with doping occurred in the overdoped case, where the low-energy spectral weight peaks shifted to the incommensurate wave vectors; see Figure 5c. This can be clearly seen in the corresponding constant-energy slices shown in Figure 6. At a low doping level, the maximal spectral weight was confined near the antiferromagnetic wave vector over a wide energy range. At a high doping level, on the contrary, low-energy excitations emerged at incommensurate wave vectors, forming a cross-like shape with the maximal spectral weight in the antinodal direction. An increase in the excitation energy led to a decrease in the incommensurability.

**Figure 7:**
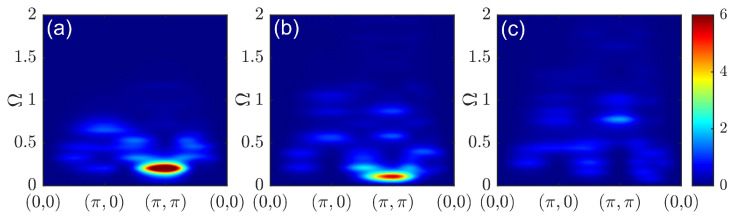
Dynamic spin susceptibility χ(q,Ω) calculated via spin-CPT for doping at p=0 (**a**), p=1/9 (**b**), and p=2/9 (**c**).

**Figure 8:**
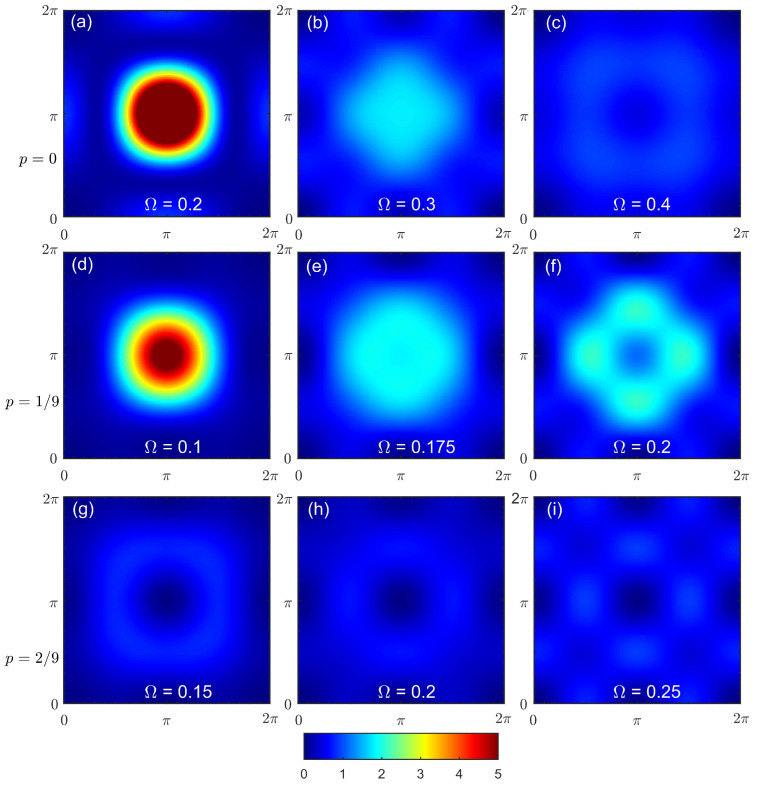
Constant energy cuts of the dynamic spin susceptibility under spin-CPT for doping at p=0 (**a**–**c**), p=1/9 (**d**–**f**), and p=2/9 (**g**–**i**) and an energy of Ω (corresponding value is shown in each panel).

The doping dependence of the spin susceptibility according to another approach, spin-CPT, is shown in Figure 7. Here, the correlation effects within a cluster were treated explicitly by the ED. The results were in qualitative agreement with the quantum Monte Carlo results for the one-band Hubbard model [26]. At half-filling, the result was substantially different from the picture provided by CPT-RPA. The response function in Figure 7a clearly resembles a spin wave spectrum. However, since only short-range correlations were present within the cluster, the maximal intensity at the antiferromagnetic wave vector shifted to higher frequencies compared to the case of a long-range order. The corresponding constant-energy cuts are shown in Figure 8 with energy values chosen to demonstrate the most prominent features for each doping. At higher energy values, the cuts revealed a dip near the commensurate wave vector (π,π), as expected for damped spin-waves and not observed for CPT-RPA. At a low doping level, the results were quite similar, but the spin response peaked less sharply at (π,π). The energy evolution of constant-energy cuts in Figure 8b,f,i shows the formation of a cross-like feature at higher energy values with the peaks of the spectral weight shifted from the center to the antinodal direction. In the overdoped regime with p=2/9, the spectral peak at (π,π) can be observed only at a high frequency in Figure 7c. All low-energy responses were at incommensurate wave vectors and were more pronounced in the antinodal direction; see Figure 8i.

Note that some typical artifacts of the cluster calculations such as size effects were present in the results. These effects should not be taken as physical results. For example, the repeating pattern barely seen in Figure 8i is typical for cluster spectral function calculations. Additionally, the momentum resolution was coarse and provided only qualitative results due to the small cluster size and the absence of intercluster interactions in the computation scheme.

**Figure 9:**
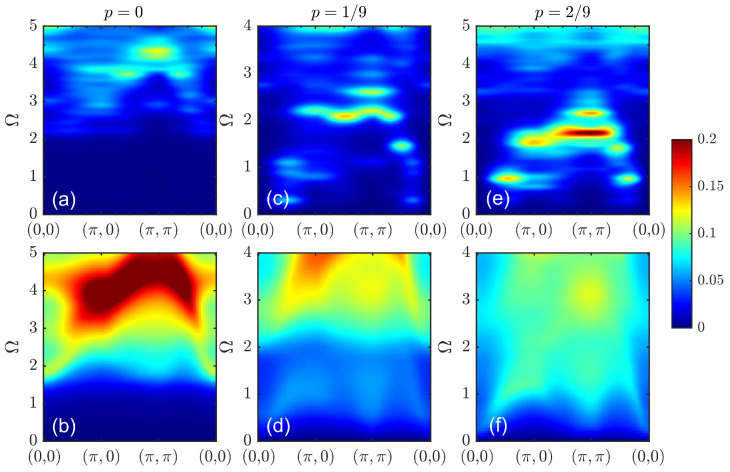
Dynamic charge susceptibility Π(q,Ω) according to charge-CPT (**a**,**c**,**e**) and CPT-RPA (**b**,**d**,**f**) for doping at p=0 (**a**,**b**), p=1/9 (**c**,**d**), and p=2/9 (**e**,**f**).

Apart from the spin susceptibility, we also calculated the dynamic charge susceptibility. This was obtained via CPT-RPA and charge-CPT; see Figure 9. For both approaches, when the doping level was increased, the spectral weight appeared at low energies. For CPT-RPA, a feature reminiscent of noninteracting susceptibility was observed, while for charge-CPT, the spectral weight below 2 eV resembled a dispersion law with the bandwidth significantly larger than that of the spin excitations. The small cluster size used herein did not allow us to elucidate complicated charge ordering effects such as those obtained via the quantum Monte Carlo method on large clusters [54]. The doping-dependent evolution of the dynamic charge susceptibility according to charge-CPT was in agreement with the most general tendencies observed using the quantum Monte Carlo approach [55,56], but the strong size effects in our calculations did not allow us to obtain more subtle features than those discussed above.

## 4. Discussion

Now, we turn to the qualitative comparison of the results obtained in the form of experimental spectra from inelastic neutron scattering (INS) on cuprates. At half-filling, cuprates are antiferromagnetic insulators due to a strong Coulomb interaction, and their spin spectrum has a spin-wave character [57]. Unfortunately, CPT-RPA failed to reproduce a spin-wave spectrum as observed in cuprates at half-filling (the spectrum obtained in this case was uninformative and is not shown). On the other hand, spin-CPT showed signatures of such a spectrum, which could be explained as stemming from the explicit inclusion of the short-range antiferromagnetic correlations within a cluster.

The addition of charge carriers into CuO${}_{2}$ planes leads to the formation of a more complicated spectrum, whose nature is still under debate [8,9]. Often, the lower downward dispersing and higher upwards dispersing branches of an hourglass spectrum are noted. These are reported to have different temperature dependencies [10] and presumably are of different natures. In the underdoped pseudogap region, several types of behavior have been found in different materials. For example, the spin spectrum of La${}_{2-x}$Sr${}_{x}$CuO${}_{4}$ has an hourglass shape in both superconducting and normal states [11], while there are no signatures of the lower downward dispersing branch in the normal state of YBa${}_{2}$Cu${}_{3}$O${}_{6+x}$ [12]. For underdoped HgBa${}_{2}$CuO${}_{4+\delta }$, there is no lower branch in either the superconducting or the normal states [13]; however, the lower branch has been observed near optimal doping in the superconducting state [14]. Both our methods in the underdoped region resulted in the signatures of the upper branch with the lowest excitation energy at (π,π) and no presence of low-energy incommensurate excitations. In particular, the absence of the low-energy incommensurate excitations for CPT-RPA, where the susceptibility was calculated from the electronic structure exhibiting pseudogap behaviour, might point to the suppression of such excitations by the pseudogap.

In the overdoped regime, both our methods produced a response at the lowest energy values around the four incommensurate wave vectors located at qualitatively similar positions to those in the INS data for overdoped La${}_{2-x}$Sr${}_{x}$CuO${}_{4}$ [58,59]. At high energy values, CPT-RPA presented a picture somewhat similar to that of INS, i.e., a weaker broad feature around (π,π). For spin-CPT, the response at and close to the (π,π) point appeared only at very high energies, although the general fact of the weakening of the high-energy response close to the (π,π) point was similar to experimental observations. A possible explanation for the observed discrepancies was that the RPA part of the CPT-RPA method underestimated the spin–spin correlations in the cuprates and thus could not completely reproduce the spin-wave-like high-energy excitations such as those seen in INS. Spin-CPT, in turn, had coarse momentum and energy resolutions and thus failed to reproduce the fine dispersive features present in the CPT-RPA results.

RIXS experiments have been successful in studying high-energy magnetic excitations, though with some restrictions on the probed area of wave vectors q. Via RIXS, magnon-like excitations in cuprates were detected, dispersing upward to energy values as high as ∼400 meV in a wide doping range from underdoped to heavily overdoped samples [1,2,3,4]. In all the spin spectra presented herein for the wave vectors in the [0,0.5π] range, mainly available in RIXS, an overall magnon-like shape of dispersion could be traced, where the highest response intensity was observed at energy values increasing as the wave vector increased from (0,0) to (0,π) and from (0,0) to (π/2,π/2). The energy width of the response was larger in the first direction than in the second. Momentum-dependent charge excitations were also found in RIXS [5]. These were dispersing upward to an energy value twice the maximal value of spin excitations, which is in general consistent with the charge susceptibility spectra obtained herein.

## 5. Conclusions

We formulated a cluster perturbation theory for two-particle correlation functions: namely, spin-CPT and charge-CPT for spin and charge dynamic susceptibilities, respectively. Both quantities were calculated by spin/charge-CPT and CPT-RPA in a wide doping range for the effective two-orbital Hubbard model obtained from the Emery p−d model. In the underdoped case, the magnetic response showed signatures of the upper branch of the hourglass dispersion and no presence of low-energy incommensurate spin excitations. Similar spectral weight distributions have been obtained using perturbation theory for strongly-correlated fermions in the normal phase [60,61,62,63]. In the overdoped case, both spin-CPT and CPT-RPA produced low-energy spin excitations located near the four incommensurate wave vectors. These results agreed with the spectra for doped cuprates obtained in INS and RIXS. The calculated momentum-dependent charge excitations were in general agreement with RIXS.

We demonstrated the crucial role of Coulomb interactions for the formation of the Fermi surface shape. A ‘bare’ band structure for the Emery model with the parameters obtained from the ab initio approach resulted in an electron-like Fermi surface, in disagreement with the ARPES data. On the contrary, the electronic correlations induced by Coulomb interactions changed the Fermi surface shape to be hole-like at a low doping level and led to the Fermi arc distribution of the spectral weight (corresponding to the pseudogap) in the underdoped region. At a high doping level, the correlation effects were shown to be small, resulting in an electron-like Fermi surface with the Lifshitz transition occurring at p≈0.13. The overall changes in the Fermi surface with doping qualitatively agreed with the ARPES data and the results of other mean-field strong coupling approaches.

One may expect that CPT-RPA would overestimate the contribution of the itinerant electrons to a two-particle quantity such as spin susceptibility. However, it is important that it considers the effect of the electronic structure, which is usually obtained quite accurately in CPT. Additionally, the electronic structure affects the spin susceptibility through the particle–hole bubble enhanced by the RPA vertex. Spin-CPT, on the other hand, converged to the exact result with an increase in the system size but underestimated the long-range correlations. In general, CPT-based approaches are simple and economic methods to calculate momentum- and energy-resolved one- and two-particle correlation functions and allow the exact consideration of short-range correlations. The latter play a key role in doped high-$T_{c}$ cuprates.

## Data Availability

Data is contained within this article.
